# Comparison of Laser Doppler Flowmetry With Contrast‐Enhanced Ultrasound to Approximate Placental Microvascular Blood Flow in the African Green Monkey (*Chlorocebus aethiops sabaeus*)

**DOI:** 10.1111/jmp.70059

**Published:** 2026-01-28

**Authors:** Yarines Gonzalez‐Rodriguez, Rachel W. Walmer, Shannon Krainiak, Kelli Carter, Kylie Kavanagh, Kennita Johnson, Sarah N. Cilvik

**Affiliations:** ^1^ Animal Resources Program Wake Forest University School of Medicine Winston‐Salem North Carolina USA; ^2^ Lampe Joint Department of Biomedical Engineering University of North Carolina‐North Carolina State University (UNC‐NC State) Raleigh North Carolina USA; ^3^ Department of Pathology Wake Forest University School of Medicine Winston‐Salem North Carolina USA; ^4^ Department of Pediatrics Wake Forest University School of Medicine Winston‐Salem North Carolina USA

**Keywords:** microvascular perfusion, nonhuman primate, OxyFlo, placenta, placental perfusion, vervet monkey

## Abstract

**Background:**

The ability to study in vivo microvascular flow within the placenta is limited. We sought to compare two techniques for evaluation of placental perfusion in a translational nonhuman primate model.

**Methods:**

We measured placental microvascular perfusion in six pregnant African green monkeys (*Chlorocebus aethiops sabaeus*) using both Laser Doppler Flowmetry (LDF) and contrast‐enhanced ultrasound (CEUS) in 2–4 discrete placental locations per animal at early‐ (EG), mid‐ (MG), and/or late‐gestation (LG). We assessed correlation between matched LDF and CEUS, with the hypothesis that we would observe concordance between these two measures of relative perfusion.

**Results:**

Using 35 total paired measurements (13 EG, *n* = 4; 12 MG, *n* = 4; 10 LG, *n* = 5), we found no correlation between LDF microvascular perfusion and CEUS microvascular flux rate (Pearson *r* = −0.2026, *R* ^2^ = 0.0411, *p* = 0.2431).

**Conclusion:**

LDF and CEUS produce discordant results with respect to placental perfusion. LDF is ill‐suited to a heterogeneous organ such as the placenta.

## 1 Introduction

The placenta is a unique, complex, and poorly understood organ that is vital to a healthy pregnancy. It is the critical interface between mother and fetus, allowing for passage and exchange of vital nutrients including glucose, oxygen, and amino acids, in addition to producing hormones that are essential to maintaining a healthy pregnancy. It is a dynamic structure that is continually growing and remodeling throughout pregnancy [1, 2, 3, 4, 5]. The placenta is a vascularly complex organ with both maternal and fetal components and a variable position and shape that makes it challenging to evaluate its function in an accurate and noninvasive manner. Techniques for invasive in vivo and longitudinal study of placental structure and function are not ethically feasible in human studies due to risks for pregnancy loss, preterm birth, and other complications [6]. This necessitates the development of new modalities to capture both regional and global placental perfusion, and highlights the ongoing need for model organisms with similar placentation and pregnancy physiology to humans, such as the nonhuman primate (NHP) [6, 7, 8, 9, 10].

Placental perfusion is a critical determinant of maternal‐fetal nutrient and oxygen transfer throughout gestation [1, 3, 11, 12, 13, 14]. As such, aberrant placental development and inadequate placental blood flow have been associated with adverse pregnancy outcomes to both mother and fetus, including intrauterine growth restriction, pre‐eclampsia, and miscarriage [3, 5, 6, 13, 15, 16, 17]. Several noninvasive modalities have been used to evaluate placental perfusion in vivo in both humans and animals, including radioactive microspheres and angiography, magnetic resonance imaging, and contrast‐enhanced ultrasound (CEUS) [1, 5, 12, 18, 19, 20, 21, 22, 23, 24, 25, 26, 27, 28, 29, 30, 31]. Utilization of many of these noninvasive techniques has been limited in both human and animal studies by cost or need for specialized equipment, or by the lack of robust safety data in humans. The paucity of cost‐effective and accessible modalities to study in vivo microvascular flow within the placenta impedes our ability to study and understand the underlying physiology in the setting of adverse pregnancy outcomes.

**FIGURE 1:**
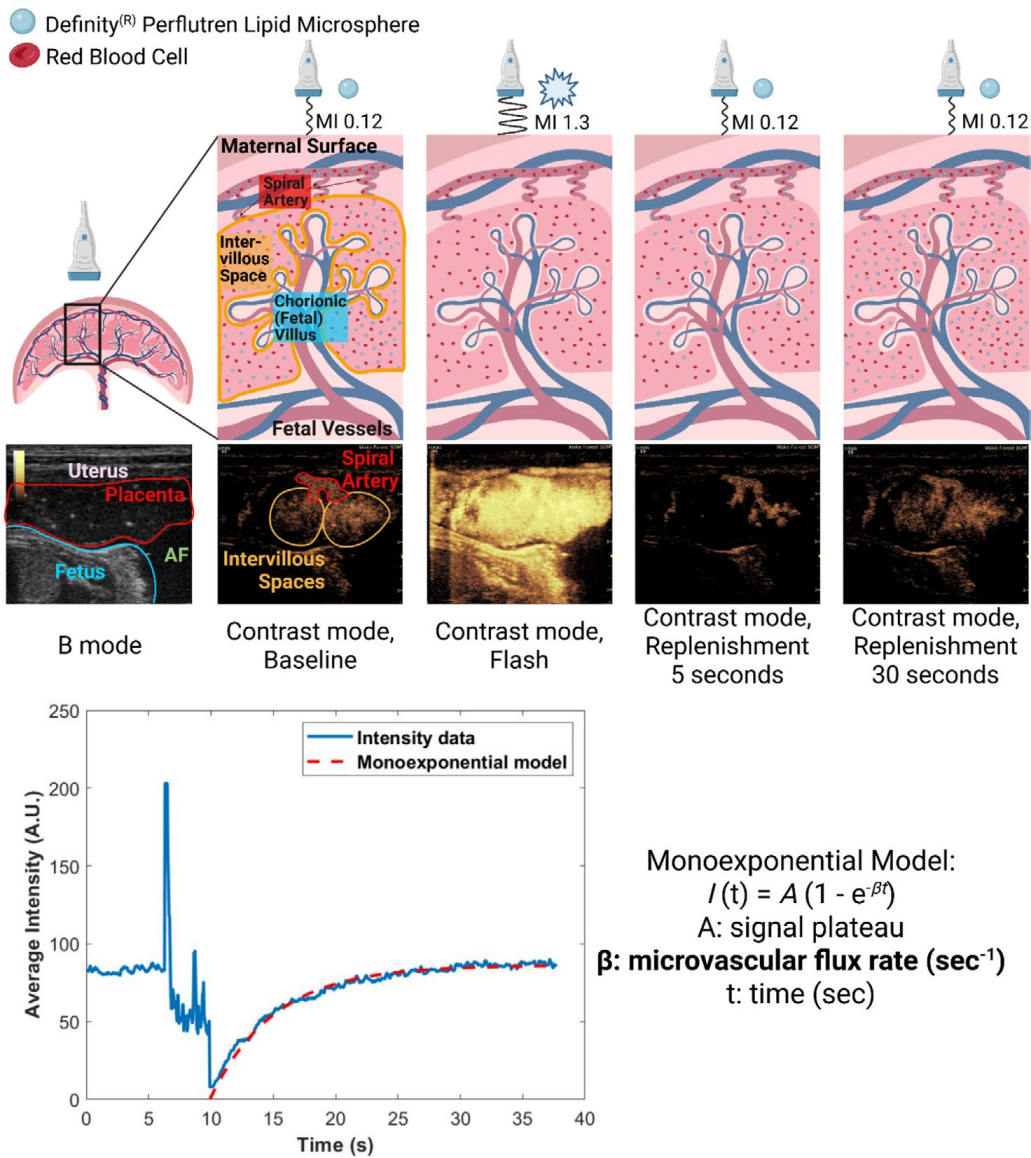
Microbubble‐based contrast‐enhanced ultrasound (CEUS). Schematic depiction of microbubble administration to enhance the microvasculature of the placenta that includes the intervillous spaces and spiral arteries. A transient increase in the mechanical index (MI) of the ultrasound results in microbubble destruction, followed by microbubble replenishment. Analysis was performed using MATLAB, with a region of interest drawn around the entire anterior placenta, as illustrated in the ultrasound image on the far left (red tracing). A time intensity curve was then generated and fit to a monoexponential model to allow for quantification of the microvascular flux rate (*β*). AF, amniotic fluid. Created in BioRender. Cilvik, S. (2026) https://BioRender.com/pg99ksd.

Microbubble‐based CEUS is a noninvasive technique that relies on the detection of gas‐filled microbubbles (< 5 μm diameter, gas core surrounded by an outer shell of lipids, proteins, or polymers) as contrast agents to provide high spatial resolution imaging and measurement of microvascular perfusion [5, 12, 18, 32, 33] (Figure 1). Microbubbles remain in the intravascular compartment and have been shown to move freely through microcirculation with a similar velocity to red blood cells [5, 33, 34]. Signal enhancement and quantitation of perfusion take advantage of the acoustic properties of microbubbles in response to different ultrasound parameters. Ultrasound evaluation at a low mechanical index of continuously infused microbubbles causes harmonic resonance resulting in an improved signal‐to‐noise ratio of the vascular compartment [5, 32]. An increase in the mechanical index results in asymmetric vibration and ultimately destruction of the microbubbles. Following return to a lower mechanical index, the reentry of microbubbles into the vascular compartment can be recorded for measurement of replenishment kinetics and allows for a quantitative assessment of tissue perfusion over time [5, 32] (Figure 1). CEUS has been used extensively for in vivo study of the NHP placenta and is an established method for evaluating microvascular perfusion in this challenging heterogeneous organ [11, 12, 13, 20, 23, 27, 35, 36, 37, 38]. However, CEUS only allows for two‐dimensional analysis of perfusion and lacks the ability to simultaneously capture the whole placenta. Therefore, this requires imaging in multiple planes to allow for a global estimation of perfusion and heterogeneity. Additionally, contrast agents are costly, which limits the number of placental areas to be evaluated per session.

**FIGURE 2:**
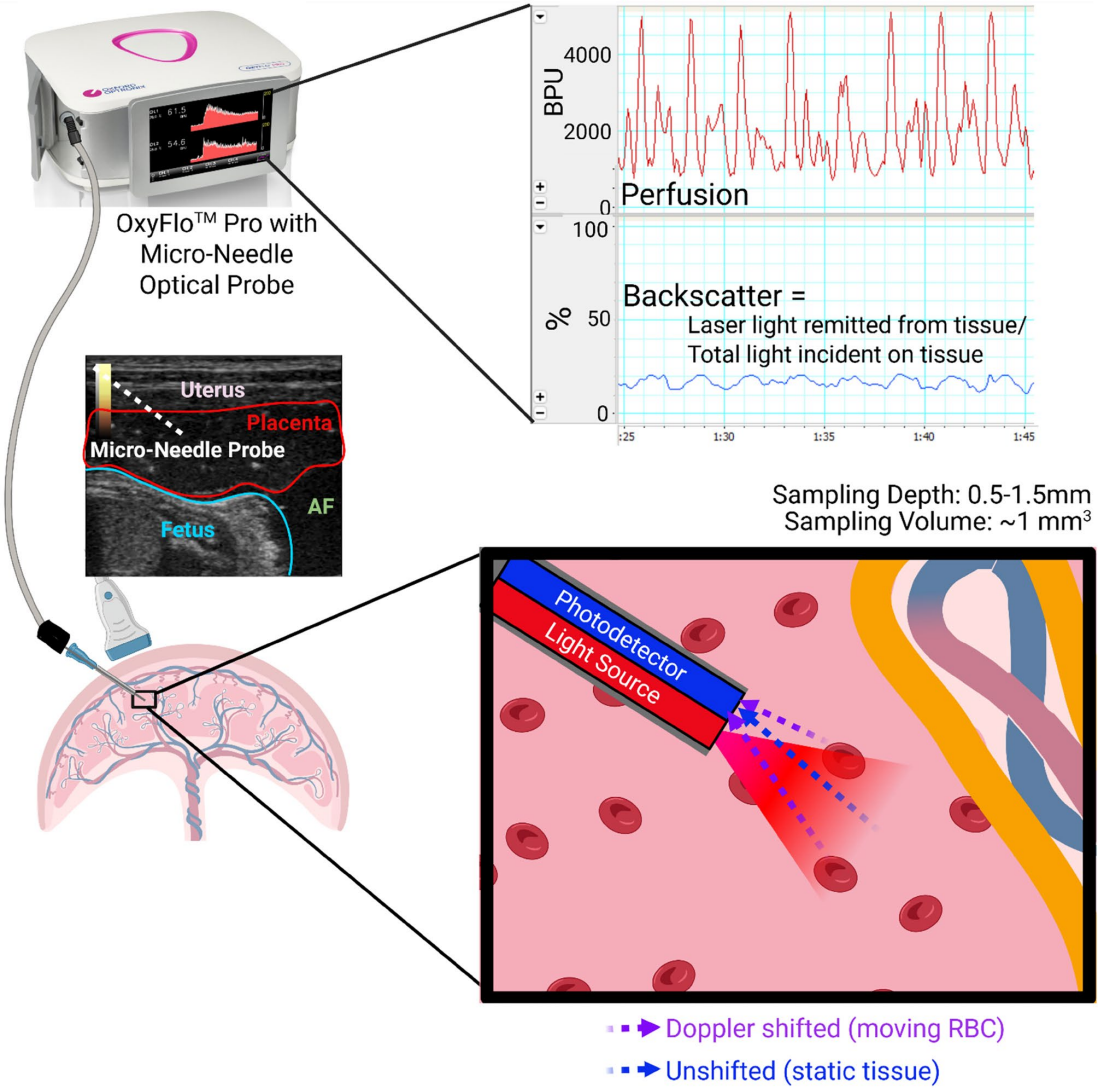
Laser Doppler flowmetry (LDF). Schematic depiction of LDF measurement of microvascular perfusion using the OxyFlo^TM^ Pro system. A reusable microneedle optical probe is inserted into the placenta under ultrasound guidance. Low power laser light is emitted from one fiber and scatters in the tissue. Light signal that encounters moving red blood cells (RBC) becomes Doppler shifted, while light that encounters static tissue remains unshifted. A second optical fiber, the photodetector, receives both the Doppler shifted (purple arrows) and unshifted (blue arrow) backscattered light. The magnitude and frequency of the Doppler‐shifted reflection is related to red blood cell velocity and red blood cell concentration in the volume of tissue under illumination from the sensor. This signal is electronically converted into an estimate of microvascular blood perfusion, measured in arbitrary blood perfusion units (BPU), as shown in the top right of the figure. AF, amniotic fluid. Created in BioRender. Cilvik, S. (2026) https://BioRender.com/1cfmlmg.

Laser Doppler flowmetry (LDF) is a minimally invasive modality that allows for the continuous measurement of microvascular blood flow in a small discrete area of tissue [38, 39, 40, 41]. A low power laser light is emitted from a fiberoptic probe and scattered within the tissue. A photodetector fiber then detects light reflected toward the flowmeter sensor. Light reflected by moving erythrocytes in the tissue becomes Doppler‐frequency shifted, while the light reflected by the surrounding tissue is not [38, 39, 40, 41]. The magnitude and frequency of the Doppler‐shifted reflection are related to red blood cell velocity and red blood cell concentration in the volume of tissue under illumination from the sensor. This signal is then electronically converted to an estimate of relative microvascular blood flow [38, 41] (Figure 2). OxyFlo^TM^ Pro LDF has been used to study peripheral vascular disease, to monitor cerebral perfusion in models of stroke and brain injury, and to evaluate tumor perfusion, angiogenesis, vital organ perfusion, and wound healing [42, 43, 44, 45, 46, 47, 48, 49, 50]. There is limited data comparing LDF with other methods of measuring tissue blood flow, particularly in a heterogeneous organ such as the placenta. However, studies have suggested that it can provide valuable information concerning the regional distribution of blood flow within an organ [40]. In contrast to CEUS, LDF requires only the initial device purchase with reusable probes, and it is able to sample more regions of tissue in less time. The OxyFlo^TM^ Pro LDF system also offers a combination of probes that would allow for simultaneous measurement of tissue oxygenation in addition to blood flow, which has the potential to provide additional important physiologic data on placental function. However, compared to CEUS, this technique is more invasive, requires anterior placental position, the sampling volume is small, and it is quite susceptible to movement artifacts.

Our research program aims to understand the development and function of the placenta longitudinally during pregnancy and in the setting of high‐risk pregnancy states. Given limitations on in vivo study of placental physiology in human studies, we are utilizing a translationally relevant NHP pregnancy model, the vervet monkey (African Green Monkey, *Chlorocebus aethiops sabaeus*). NHPs have been widely used as models to evaluate the uteroplacental pathology and placental perfusion due to their similar gestational development and placental structure to humans [6, 7, 8, 9, 10]. While CEUS has been used extensively to study placental microvascular perfusion in NHPs, LDF has never been tested in the placenta to our knowledge. The approximately 4–6 kg pregnant female vervet frequently has at least a portion of the placenta present anteriorly, and it is easily accessible with a 5‐cm probe. Therefore, the aims of this study were to determine if OxyFlo^TM^ Pro LDF was a safe and feasible method to quantify placental perfusion, and to assess its accuracy with comparison to the well‐established CEUS imaging for evaluation of relative microvascular perfusion of the placenta. We hypothesized that LDF would be technically feasible and safe in the vervet, and that estimates for microvascular blood flow would correlate with CEUS in matched areas of the vervet placenta. The ability to use LDF, and specifically the OxyFlo^TM^ Pro system, would ultimately allow for more robust data collection on placental perfusion for significantly lower cost, while opening the possibility of combining perfusion assessments with measurement of tissue oxygenation, another critical measure of placental function and pregnancy health.

## 2 Materials & Methods

### 2.1 Humane Care Guidelines

The authors confirm adherence to the ethical policies of the journal, as noted on the journal's author guidelines page. We performed all animal studies at Wake Forest University School of Medicine (WFUSM), which is accredited by the Association for the Assessment and Accreditation of Laboratory Animal Care (AAALAC). Studies were conducted following protocol approval by the WFUSM Institutional Animal Care and Use Committee (protocol #A21‐022). All animal procedures conformed to the requirements of the USDA Animal Welfare Act and complied with international, national, and institutional guidelines for humane animal treatment, including those set forth by the US National Research Council [51] and the US Public Health Service “Policy on Humane Care and Use of Laboratory Animals” and “Guide for the Care and Use of Laboratory Animals.”

### 2.2 Subject Selection, Animal Care, and Study Timepoints

**FIGURE 3:**
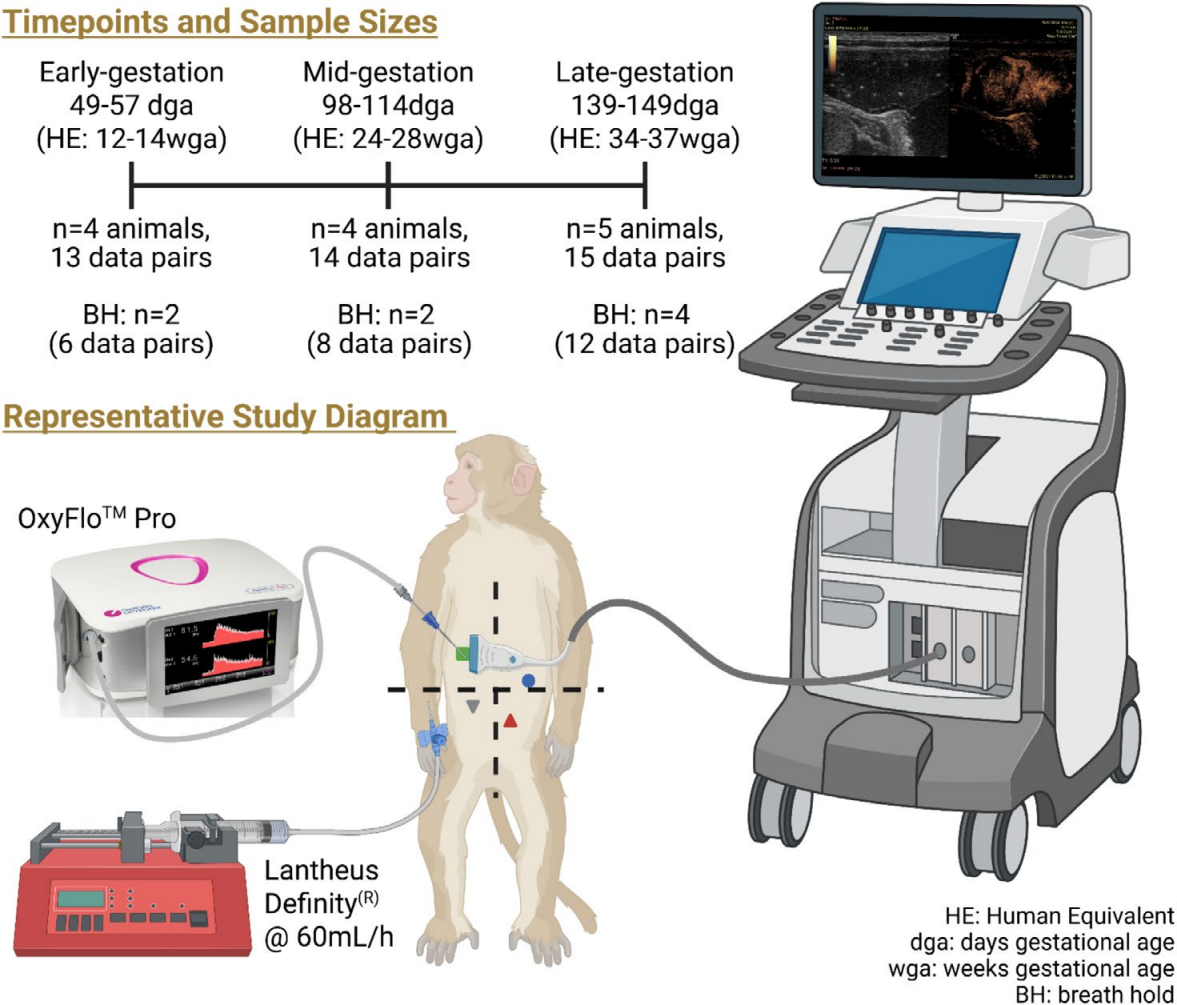
Study design for comparison of contrast‐enhanced ultrasound and laser Doppler flowmetry using OxyFlo Pro. We studied pregnant female vervets longitudinally at early‐ (49–57 days gestational age; dga; 0.3–0.35 gestation), mid‐ (98–114 dga; 0.6–0.7 gestation), and late‐gestation (139–149 dga; 0.85–0.9 gestation); samples sizes are representative of the full data set before outlier removal. Animals subjected to breath holds (BH) during LDF measurements are noted on the bottom row of the timeline, with number of breathing vs. breath hold matched data pairs in parentheses. Bottom figure showing a representative study diagram with 4 discrete placenta positions illustrated by different shapes (green square, gray triangle, red triangle, blue circle). We performed CEUS first, followed by LDF to collect matched data pairs at each anterior placental location. HE, human equivalent gestation; wga, weeks gestational age. Created in BioRender. Cilvik, S. (2026) https://BioRender.com/hjh9gw1.

The overall study design is depicted in Figure 3. We selected 6 historically healthy (weight < 6.5 kg, normotensive, nondiabetic), early‐gestation (< 45 days gestation; dga) pregnant adult female African Green monkeys (vervets; *Chlorocebus aethiops sabaeus*) between the ages of 5 and 12 years from the multigenerational pedigreed Vervet Research Colony (VRC) at WFUSM. We dated pregnancies according to established imaging and dating protocols in vervets [52, 53]. The St. Kitt's‐descended VRC houses monkeys according to matrilineal social groups (16 total), with one intact adult male included in each group. Each corral contains both indoor and outdoor space containing perches, platforms, climbing structures, and other enrichment objects [52]. All animals had *ad libitum* access to food (commercial standard laboratory monkey chow, Lab Diet 5038; Purina, St. Louis, MO) and water apart from fasting required for study days. They received additional supplementation five days a week with fresh fruits and vegetables and other enrichment foods such as popcorn.

We performed the studies described below at two or three timepoints per pregnancy in each female: early gestation (49–57 dga; EG; *n* = 4), mid‐gestation (98–114 dga; MG; *n* = 4), and/or late gestation (139–149 dga; LG; *n* = 5) (Figure 3), with full term being approximately 157–168 dga [52, 53, 54, 55]. All six females selected had a history of at least two prior successful pregnancies with liveborn infants. For all studies, monkeys were fasted for at least 12 h with continued access to water. VRC staff corralled monkeys into capture tunnels, where we sedated them with intramuscular ketamine (15 mg/kg) and transported them to a surgical suite. We intubated the monkeys, anesthetized them with 1%–2% inhaled isoflurane gas, and maintained them on invasive ventilation throughout the study. We placed a 22‐gauge IV catheter and provided maintenance IV fluids with 0.9% saline and 5% dextrose at 5 mL/kg/h. Our team continuously monitored blood pressure, heart rate, respiratory rate, perfusion, and end‐tidal carbon dioxide and recorded vital signs every 10 min throughout the procedure, with ventilator and anesthesia adjustments made as clinically indicated to maintain normal vitals. We positioned monkeys in dorsal recumbency and shaved the abdomen of each animal. We aseptically prepared the abdomen by alternating chlorhexidine and alcohol embedded gauzes and then placed sterile surgical drapes on the abdomen and a sterile cover on the ultrasound probe.

### 2.3 Contrast‐Enhanced Ultrasound (CEUS)

We performed CEUS using the GE Logiq S8 equipped with a 5–12 MHz linear array probe (GE 11L) to evaluate regional two‐dimensional placental perfusion using the commercially‐available DEFINITY^(R)^ microbubble contrast agent (Lantheus Medical Imaging), as described extensively in the rhesus macaque placenta [5, 11, 13, 27, 35, 56, 57, 58, 59] and illustrated in Figure 1. We activated microbubbles with 45 s of vigorous shaking in a Vialmix according to manufacturer instructions, then diluted to a 5% solution in 0.9% saline (1 mL DEFINITY^(R)^ + 19 mL saline). We infused the microbubble solution into the pregnant vervets at 1 mL/min to evaluate microvascular perfusion within the placenta using contrast mode on the ultrasound system. Once microbubble signal intensity reached steady state, we located a placental cotyledon with intervillous space supplied by a maternal spiral artery and maintained the probe in a fixed position. We captured baseline microbubble signal (mechanical index 0.12) for 5 s prior to acquiring a 30‐second flash‐replenishment sequence. During the flash sequence, the mechanical index temporarily increased to 1.3 for 60 frames to destroy bubbles in the transducer field of view, then returned to 0.12 for the remainder of the recording to measure microbubble reentry into the intervillous space (Figure 1). We captured two replicate recordings at each location. We evaluated 4 distinct placental positions in each animal per timepoint. All imaging was performed by a single person (SNC). Contrast mode imaging parameters were set as follows for each video recording: frame rate 23 frames per second, mechanical index 0.12, gain 41 dB, dynamic range 75 dB, imaging depth 4 cm, and transducer focal point 3.2 cm.

We exported CEUS scans as DICOMs for offline processing and analysis by a blinded team member (RW) using MATLAB (MathWorks). She placed a region of interest over the entire visible anterior placenta, as shown in Figure 1, and extracted average microbubble signal across time [*I(t)*]. She fit time‐intensity curves for reperfusion using a mono‐exponential model, $I\left(t\right)=A\left(1-e^{-\mathrm{\beta t}}\right)$, to extract parameters related to blood volume (A, signal plateau), velocity (β, flux rate), and flow (Aβ) [60]. Microvascular flux rate (β) is the rate of spiral artery or intervillous space refill until signal saturation is achieved, as reviewed by Roberts and Frias [5]. The mono‐exponential model goodness of fit was evaluated using the adjusted *R* ^2^ value calculated in MATLAB. Weak fits (*R* ^2^ < 0.3) were removed and only extracted parameters from model fits with *R* ^2^ > 0.3 were included in the final analysis. We calculated the mean β, standard deviation, and coefficient of variation (CV) from CEUS replicates at each placental position; median CV for imaging replicates was 10.64% (range 0.15%–41.55%).

### 2.4 Laser Doppler Flowmetry (LDF)

We performed LDF (OxyFlo^TM^ Pro; Oxford Optronix, UK) at each anterior placental location (2–4 positions per monkey per timepoint) following completion of replicate CEUS video capture, as shown in Figure 2. We paused microbubble infusion, transitioned from contrast to standard B‐mode imaging on the ultrasound system, and maintained the ultrasound transducer in a fixed location. We sterilely inserted a 22G angiocatheter into the approximate placental area of visualized flow from the preceding CEUS imaging. After removal of the needle, we advanced a custom reusable 50 mm × 1 mm (length × diameter) blunt needle blood flow probe (Oxford Optronix, UK) through the indwelling catheter and into the placenta. We recorded real‐time relative perfusion (blood perfusion units; BPU) using the integrated LabChart8 software. We identified a 30‐second period of stable signal for each placental position and extracted the LDF mean, median, minimum, maximum, and standard deviation. In a subset of the monkeys (4 individual monkeys, 26 discrete placental positions + timepoints), we performed two 10‐second breath holds at each placental location to determine whether motion altered the accuracy of the relative perfusion measurements. Monkeys received buprenorphine (0.02 mg/kg IM) at the conclusion of LDF measurements and before cessation of isoflurane anesthesia. Once awake with active swallowing, we extubated the monkeys, allowed them to fully recover from anesthesia in single cages, and then returned them to their social housing to allow for continuation of pregnancy and natural delivery of offspring.

### 2.5 Statistical Analysis

We repeated measurements in five of the six females at two gestational timepoints, and repeated measurements in one female at all three gestational timepoints. Missing timepoints were the result of broken equipment or inability to purchase contrast agent due to supply issues. We made a total of 42 time‐ and location‐matched LDF and CEUS paired measurements. We did not include CEUS measurements of posterior placental locations without a matching LDF measurement; we will publish these complete CEUS data as part of a larger study in the future. We used GraphPad Prism 10.5.0 for all analyses. We evaluated for outliers using the ROUT method (Robust regression and OUTlier removal) with Q = 1% and removed any pairs containing an outlier from further analysis [*n* = 7 pairs (5 LG, 2 MG) removed]. We then compared the mean microvascular flux rate (β, sec^−1^) from replicate CEUS measurements against the corresponding 30‐second median LDF microvascular perfusion (BPU) using Pearson correlation analysis to evaluate the remaining 35 paired measurements, with *p* < 0.05 indicating a significant correlation between these measurements and the Pearson correlation coefficient (*r*) representing the strength and directionality of the relationship. Given the heterogeneity of the placenta with known remodeling throughout gestation, each location‐ and time‐matched pair was treated as independent. To determine whether the gestational timepoint affected the concordance between measurements, we performed a separate Pearson correlation analysis by gestational age (EG *n* = 13 pairs; MG *n* = 12 pairs; LG *n* = 10 pairs). Finally, to determine whether movement related to respiratory effort affected LDF measurements, we performed Pearson correlation analysis to compare time‐ and location‐matched LDF measurements with ventilator‐assisted breathing (30‐second LDF median value) and with breath holding (average of two LDF median values each with 10‐second breath hold) (*n* = 26 pairs).

## 3 Results

**FIGURE 4:**
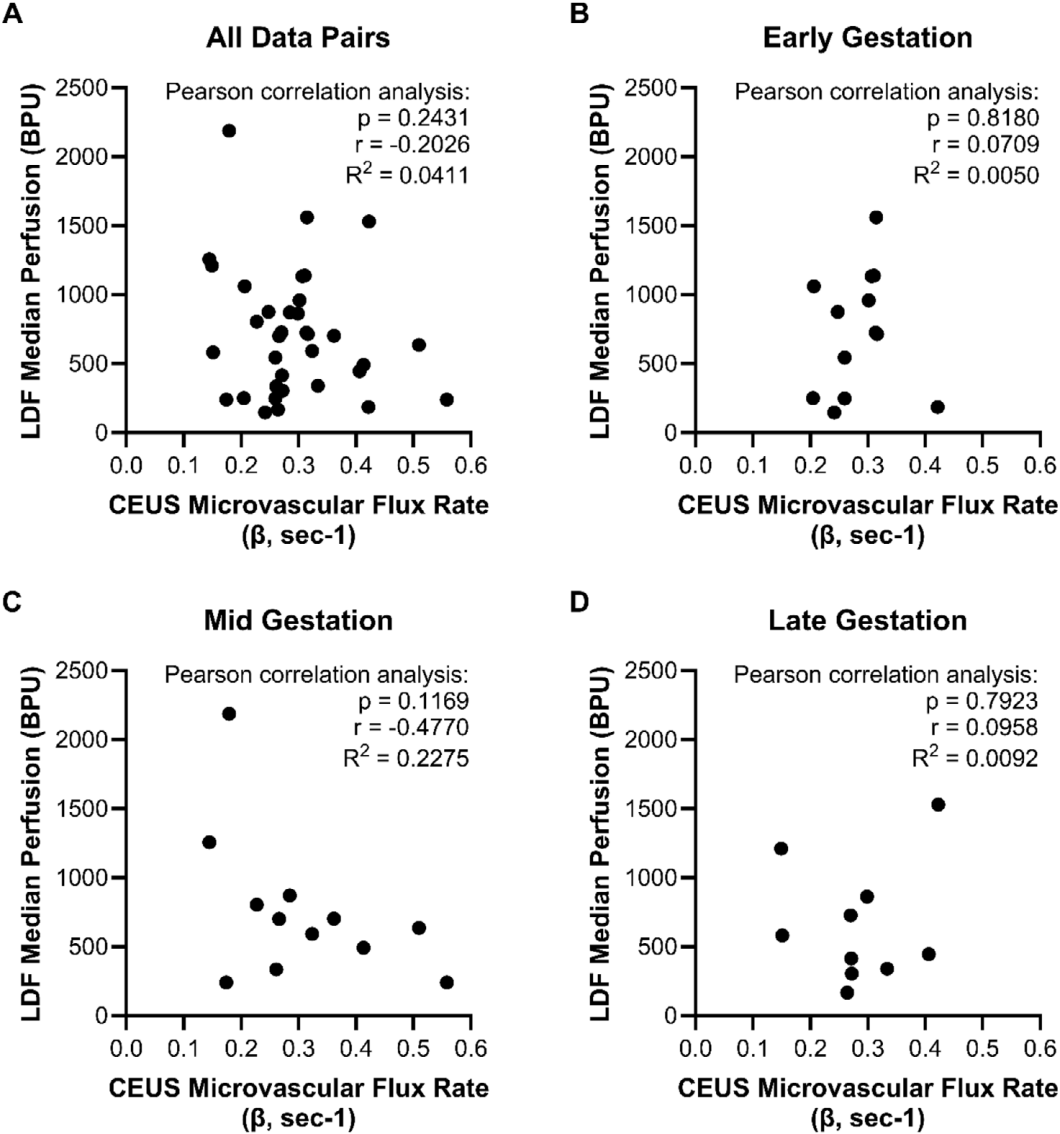
No correlation between LDF and CEUS measurements of microvascular placental perfusion. Each data point represents a time‐ and position‐matched measurement of median LDF (*y*‐axis; median value over 30‐s of continuous recording) vs. CEUS (*x*‐axis; mean *β* value of 2 replicates), with results of Pearson correlation analysis included for each graph. No correlation was observed when comparing all data across all gestational time points (A; *n* = 35 pairs from 6 females). Similarly, there was no correlation between LDF and CEUS measurements at any gestational time point (B, early gestation, *n* = 13 pairs from four females; C, mid gestation, *n* = 12 pairs from four females; D, late gestation, *n* = 10 pairs from five females).

There was no correlation between placental microvascular perfusion measured by CEUS (*β*) and OxyFlo^TM^ Pro LDF (Pearson correlation: *p* = 0.2431, *r* = −0.2026, *R* ^2^ = 0.0411, *n* = 35 paired LDF‐CEUS measurements, 7 outlier pairs removed), regardless of the gestational timepoint (EG: *p* = 0.8180, *n* = 13 pairs; MG: *p* = 0.1169, *n* = 12 pairs; LG: *p* = 0.7923, *n* = 10 pairs) (Figure 4). As *β* represents the microvascular flux rate (velocity), we also compared microvascular flow (A × *β*) from CEUS with LDF, and similarly found no correlation between the two measurements (Pearson correlation: *p* = 0.3898, *r* = 0.1456, *R* ^2^ = 0.0212, *n* = 37 paired LDF‐CEUS measurements, 5 outlier pairs removed; data not shown).

**FIGURE 5:**
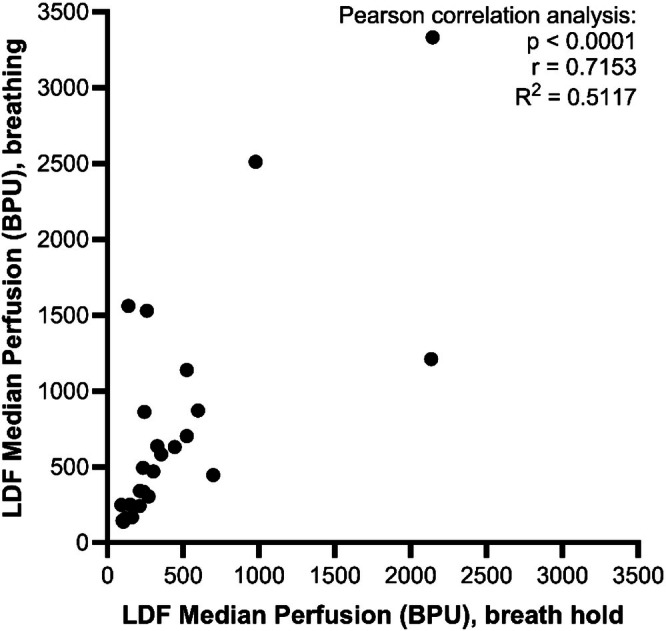
Strong correlation between paired LDF measurements with or without respiratory motion. *X*‐axis represents the average median value of two replicate 10‐s recordings while performing a breath hold. *Y*‐axis represents the median value of a 30‐s LDF recording with continuous ventilator‐assisted breathing. Each data point represents a time‐ and location‐matched LDF recording (*n* = 26 paired readings from four monkeys). Results of Pearson correlation analysis included on graph.

To determine whether motion artifact from respiratory effort was a contributing factor for the lack of correlation between CEUS and LDF, we used a subset of the animals (*n* = 4) to compare the median LDF microvascular perfusion with and without a breath hold. We found a strong positive correlation between LDF measurements with or without motion (Pearson correlation: *p* < 0.0001, *r* = 0.7153, *R* ^2^ = 0.5117, *n* = 26 paired LDF measurements) (Figure 5), despite a larger coefficient of variation in tracings with respiratory motion (median CV breathing 32.8% vs. median CV breath hold 15.1%).

## 4 Discussion

The primary goal of our study was to evaluate the feasibility and accuracy of an existing technology to measure microvascular perfusion (LDF using OxyFlo^TM^ Pro system) in a novel tissue, the placenta. We hypothesized that LDF would produce perfusion results that correlated with a validated imaging modality, CEUS, for measurement of microvascular flux rate within the NHP placenta. If validated, the OxyFlo^TM^ Pro LDF system would provide a more cost‐effective and efficient measurement of placental perfusion in our translationally relevant NHP pregnancy model. Both techniques were safe, feasible, and well‐tolerated methods to evaluate in vivo placental microvascular perfusion in the vervet monkey (African Green Monkey, *Chlorocebus aethiops sabaeus*). There were no adverse events reported after the procedures and all animals successfully delivered liveborn healthy infants. Unfortunately, LDF measurements showed no correlation with CEUS microvascular flux rates in the placenta.

There are several reasons that LDF failed to accurately measure placental perfusion. According to the manufacturer (Oxford Optronix), the typical sampling volume for the OxyFlo^TM^ Pro probe is approximately 1 mm^3^. In a complex vascular organ such as the placenta with both maternal and fetal blood supplies, nonuniform vascular distribution, and heterogeneous blood flow, this small sampling volume would be subject to large variation in BPU with even small changes in probe position. We hypothesized that some of the discordance between the LDF and CEUS measurements may have been the result of respiratory motion on the LDF signal. However, evaluation of perfusion with LDF during breath holds was strongly correlated to LDF during mechanical ventilation. Therefore, it is more likely that the precise probe positioning was not an accurate representation of the flow within that placental region. Under standard B‐mode ultrasound guidance, one cannot identify areas of perfusion to guide probe placement to the intervillous spaces for targeted and precise measurement of perfusion. In contrast, CEUS provides direct visualization of perfusion allowing for measurement of flux rate within a region of interest. While we attempted to insert the probe into the intervillous space identified on CEUS imaging, we could not perform these assessments simultaneously and thus could not ensure precision with the placement of the tissue probe. Given the very small sampling volume of the OxyFlo^TM^ probe, we would recommend extreme caution in use and interpretation of LDF for measurement of perfusion in a large vascularly heterogeneous tissue such as the placenta.

One could envision that LDF has potential utility in measuring acute changes in blood flow in a localized placental area in response to interventions, such as changes in body temperature, oxygenation, hemoglobin concentrations, blood pressure, and more. The probe could be inserted into a particular area to measure real‐time perfusion from baseline to post‐intervention, similar to previous publications that have measured perfusion changes in the skin, skeletal muscle, or brain [46, 47, 49, 61, 62, 63]. However, as mentioned previously, this would only provide perfusion data on 1 mm^3^ of the placental volume and would not be representative of the physiological changes throughout the tissue, so the utility of this approach in any vascularly heterogeneous tissue is also questionable.

Our study does have some limitations. Most notably, because we were unable to ensure that the LDF measurements were taken from the highly perfused region visualized on CEUS, we broadened our ROI measurements to encompass the entire region of the placenta imaged at that position rather than limiting it to the area of high signal within the intervillous space. This choice was made after preliminary analysis of the first 2 subjects demonstrated large discrepancies in relative perfusion between the 2 techniques when using ROIs targeted only to the intervillous space (not shown). Furthermore, we had intended to perform longitudinal measurements at 3 timepoints in all subjects to also evaluate heterogeneity of perfusion within the placenta over time as measured with both techniques. However, due to OxyFlo^TM^ probe malfunction and supply issues with DEFINITY^(R)^ microbubble contrast agent, there were several time points missing paired data collection. Given the clear lack of concordance between the two measurements, we determined that further use of the OxyFlo^TM^ Pro system was of little utility. We are currently evaluating regional heterogeneity in perfusion using a larger data set of longitudinal CEUS.

## 5 Conclusion

LDF using a needle probe inserted into the placenta is feasible in the vervet monkey, but microvascular perfusion measurements do not correlate with those assessed with the validated CEUS technique. This is likely secondary to a limitation in sampling volume that does not permit accurate assessment of a heterogeneously perfused tissue like the placenta. Furthermore, LDF is unable to access posterior placental tissue, limiting its ability to globally assess perfusion within the placenta. While LDF would be a more economical method for measuring perfusion in animal models compared to CEUS, its technical limitations and questionable accuracy negate any utility in the placenta. The placenta continues to be a challenging tissue to study, and NHPs, such as the vervet monkey, remain essential as the ideal translational model to longitudinally evaluate placental physiology through the development of new imaging and sampling techniques.

## Funding

Research reported in this publication was supported by the National Heart, Lung, and Blood Institute of the National Institutes of Health (NIH) under award numbers R56HL164434/R01HL164434 (SNC), the National Institute of General Medical Sciences of the NIH under award number IRACDA 2K12GM000678‐24 (RW), as well as the US Department of Defense (DoD) under award number W81XWH‐21‐1‐0565 (KK). The Vervet Research Colony at WFUSM is supported as a biomedical resource by the NIH Office of the Director under award number P40OD010965 (M. Jorgensen). The content in this manuscript is solely the responsibility of the authors and does not necessarily represent the official views of the NIH or DoD.

## Ethics Statement

The authors confirm adherence to the ethical policies of the journal, as noted on the journal's author guidelines page. All animal studies were performed at Wake Forest University School of Medicine (WFUSM) and complied with international, national, and institutional guidelines for humane animal treatment, including those set forth by US National Research Council “Guide for the Care and Use of Laboratory Animals” and the US Public Health Service “Policy on Humane Care and Use of Laboratory Animals” and “Guide for the Care and Use of Laboratory Animals.” All studies were conducted following approval of the WFUSM Institutional Animal Care and Use Committee (protocol #A21‐022).

## Conflicts of Interest

The authors declare no conflicts of interest.

## Data Availability

The datasets used and/or analyzed during the current study are available from the corresponding author on reasonable request.
